# Supplementary material for: Changes in the Epidemiology of Diabetic Retinopathy in Spain: A Systematic Review and Meta-Analysis
Source: Healthcare (Basel). 2022 Jul 16;10(7):1318. doi: 10.3390/healthcare10071318 (PMC9320037; doi:10.3390/healthcare10071318)
Supplement: Supplementary file 1 [file healthcare-10-01318-s001.zip › healthcare-1759650-supplementary.pdf]

healthcare

MDPI

Supplementary material

Literature research and study selection

Following the I guideline of Preferred Reporting Items for Systematic Reviews and Meta-Analyses (PRISMA) [12], we conducted a systematic review of the literature in order to identify all relevant publications on the prevalence and incidence of DR in Spain.
Database.
A general data search included the following databases EMBASE, Web of Science, Scopus, and MEDLINE, complemented by Google Scholar search.
We search first in Medline for the following words, diabetes mellitus, diabetic retinopathy, screening, prevalence and/ or incidence of diabetic retinopathy, in English and their equivalent descriptions in Spanish languages. Also, we search all the different combinations of those keywords to find the maximum possible number of citations. The search was carried out between the years 2001 and 2020. Relevant articles were original reports of population-based studies restricted to selected in Spain for study and Western countries (Europe, USA, Canada, Australia) for discussion. and designed to specifically describe the prevalence and/or incidence of diabetic retinopathy. The same search was carried out in Embase, Scopus, web of science and google scholar.
The next step was to remove the cross references between the different databases. in the end they stayed 174 records in Medline, then we compared the results with Embase (165 records), Scopus (172 records) web of science (87 records) and google scholar (198 records). First, we eliminate those records that were only abstracts, letters or reviews. Next, we revised all records and compare titles and authors, eliminating those records that were the same in the different databases. Then, we are left with a total of 90 relevant records. After this initial database search, abstracts were examined and full texts underwent further evaluation for eligibility by two reviewers (PRA and MBB). Any ambiguities or disagreements between reviewers were resolved through discussion with the senior researcher (JFB).
We removed 13 records for duplication and 13 for other reasons (studies made before 2001 but published later). A second step excluded 38 records that do not meet the requirements of the inclusion criteria. Third step excluded 2 records that no include all required epidemiological data. Finally, we search 31 records that meets inclusion and exclusion criteria from databases.
Independently we search other records and we encountered 2 records from web sides and 5 from public organization (Ministry of health and dependent agencies).
All remain records are revised, and authors were contacted where needed. In fact, the most important records for the study have been made by the other authors of this study (ML, MAMB, APR, SA, JFN and JA). Some studies reported non-weighted prevalence and incidence data. Where both weighted and non-weighted prevalence and incidence data were reported, we used the non-weighted data to ensure consistency and allow for comparison.
At the end of the process, 38 articles, doctoral theses and documents that met the inclusion and exclusion criteria were selected. For cross-sectional study we used nine articles and a thesis. For longitudinal study we used five articles and a doctoral thesis. We used sixteen articles for descriptions of epidemiology and screening of diabetic retinopathy. Finally, we included seven documents that had been published by the Ministry of Health and Regional health care systems.
